# Purification Effect of PES-C Ultrafiltration Membrane Incorporated with Emodin on Acanthopanax Senticosus Injection

**DOI:** 10.3390/ph16081135

**Published:** 2023-08-10

**Authors:** Jie Zhang, Chunyan Zhang, Hongdan Xue, Chengbo Lu, Rong Rong, Jinjing Li, Shujing Zhou

**Affiliations:** 1 Heilongjiang Provincial Key Laboratory of New Drug Development and Pharmacotoxicological Evaluation, Jiamusi University, Jiamusi 154007, China; chunyzhang2023@163.com (C.Z.); lcb220310@163.com (C.L.); dryang1988@163.com (R.R.); m13846184696@163.com (J.L.); 2 HeBei University of Architecture, Zhangjiakou 075000, China; xuehd0725@163.com

**Keywords:** ultrafiltration membrane, PES-C, emodin, acanthopanax senticosus injection, adverse reactions

## Abstract

A new PES-C/emodin ultrafiltration membrane was prepared by blending natural emodin with phenolphthalein polyethersulfone (PES-C) and was used to purify an acanthopanax senticosus injection in this study. Regarding the purified acanthopanax senticosus injection, its color became lighter, and its clarity increased. On the contrary, for an acanthopanax senticosus injection containing macromolecules, its color deepened, and its turbidity increased. Thermal stability of the purified acanthopanax senticosus injection was the best, followed by the original solution of the acanthopanax senticosus injection, and the acanthopanax senticosus injection containing macromolecules was the worst. The fingerprint spectrum of the purified acanthopanax senticosus injection was similar to the original solution of the acanthopanax senticosus injection, the relative peak area of each single peak was greater than 0.95, and the relative peak area of the total peak was greater than 0.96. Compared with the original solution of the acanthopanax senticosus injection, the histamine release amount and cell degranulation rate of the acanthopanax senticosus injection containing macromolecules increased, while those of the purified acanthopanax senticosus injection decreased, which reduced the risk of allergic reaction to a certain extent. “Inverse proof” confirmed that the acanthopanax senticosus injection containing macromolecules had certain liver and kidney toxicity, which indirectly proved that the liver and kidney toxicity of the purified acanthopanax senticosus injection was effectively reduced.

## 1. Introduction

An acanthopanax senticosus injection is a single preparation processed from acanthopanax senticosus through “water extraction and alcohol precipitation”, which is widely used in the treatment of cerebrovascular diseases, coronary heart disease, angina pectoris, acute cerebral infarction, and other diseases [1]. However, with the continuous use of the acanthopanax senticosus injection in clinical practice, the occurrence of adverse reactions (ADR) also increases; the common adverse reactions include systemic damage, skin damage, etc. [2]. One of the reasons is that the ineffective components remaining in acanthopanax senticosus injections, such as protein, condensed tannin, and pyrogen, are not completely removed, which leads to the generation of adverse reactions. Methods for removing macromolecules from traditional Chinese medicine injections generally have the disadvantages of high energy consumption, low efficiency, and high pollution, while the ultrafiltration technology is favored for its advantages of high separation efficiency and strong continuity.

The separation efficiency of ultrafiltration technology depends on the membrane materials. Common membrane materials include organic polymer and inorganic materials, among which the former is widely used. Organic polymer membrane materials mainly include polysulfone [3,4,5], cellulose [6,7], polyamide [8,9], fluoropolymers, etc [10,11,12]. Phenolphthalide polyethersulfone (PES-C) is a kind of polysulfone, its structure determines its good hydrolysis resistance, mechanical property, and thermodynamic stability, and it is widely used in the preparation of fuel cell membranes [13], ultrafiltration/nanofiltration membranes [14,15] and nanofiber composite membranes [16]. However, due to the inherent hydrophobic property of PES-C polymer, its service life is seriously affected. Therefore, the hydrophilic modification of the PES-C membrane is imperative. There are many modification methods for membrane materials, among which blending modification is the most widely used.

In recent years, natural products and their derivatives have gradually attracted researcher attention because of their decontamination and purification functions. Studies have shown that emodin isolated from the root of polygonum cuspidatum has antibacterial, anti-inflammatory, and other biological activities [17,18,19]. Emodin has an obvious inhibitory effect on gram-positive bacteria such as Staphylococcus aureus. At the same time, the structure of emodin contains a certain number of hydroxyl groups, which lays a theoretical foundation for improving the hydrophilic and anti-pollution properties of the membrane, and it is expected to have a good application prospect in the membrane field.

In this paper, with emodin as an additive, a PES-C/emodin ultrafiltration membrane with excellent comprehensive performance was obtained. It was used to purify the acanthopanax senticosus injection to reduce the risk of adverse reactions.

## 2. Results and Discussions

According to the preliminary experimental results [20], the best comprehensive performance of the prepared PES-C/emodin ultrafiltration membrane was as follows: filtration performance showed a water flux of 387.41 L∙m^−2^∙h^−1^, and a rejection rate of 99.83%; Hydrophilic had a water content of 6.78%, and a contact angle of 65.71°; Anti-pollution performance had a flux recovery rate of 57.05%, and a BSA adsorption rate of 1.44% and the antibacterial diameter of 2.30 cm on S. aureus. Moreover, compared with the PES-C ultrafiltration membrane, the optimal PES-C/emoin ultrafiltration membrane significantly reduced bacterial adhesion.

**Figure 1:**
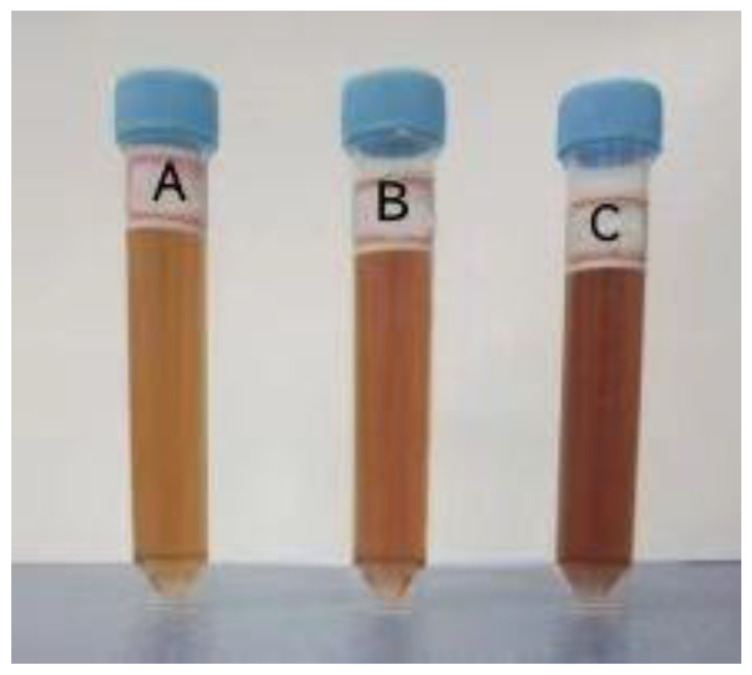
Effect pictures of appearance quality of purified acanthopanax senticosus injection, acanthopanax senticosus injection, and acanthopanax senticosus injection containing macromolecule compounds. Note: (A) Purified acanthopanax senticosus injection; (B) Original solution of acanthopanax senticosus injection; (C) Acanthopanax senticosus containing macromolecules.

Preparation and appearance color detection results of the purified acanthopanax senticosus injection, acanthopanax senticosus injection, and acanthopanax senticosus injection containing macromolecules are shown in Figure 1. From Figure 1, compared with the original liquid of acanthopanax senticosus injection, the color of the purified acanthopanax senticosus injection was slightly lighter, and its clarity was improved. For the acanthopanax senticosus injection containing macromolecules, although there was no precipitation, its color was significantly dark, indicating that treatment of PES-C/emoin ultrafiltration membrane can effectively improve the clarity of an acanthopanax senticosus injection.

**Figure 2:**
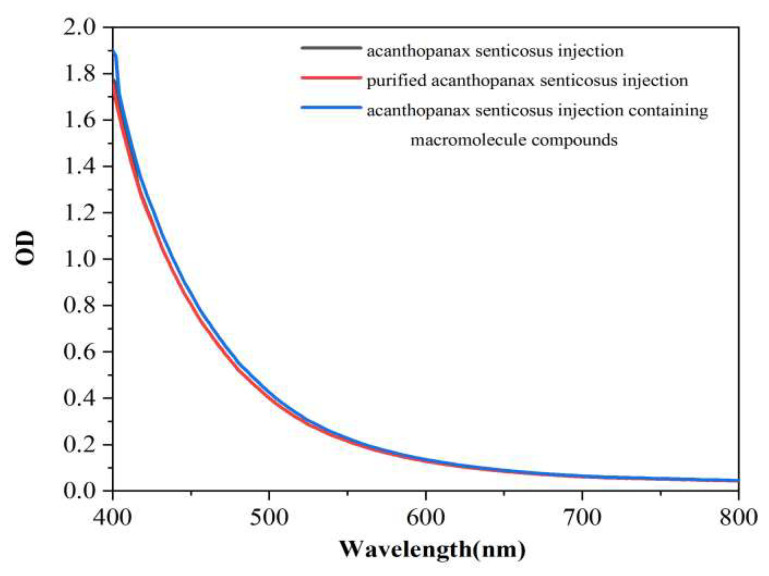
Thermal stability absorption curve of injection (0 d).

**Figure 3:**
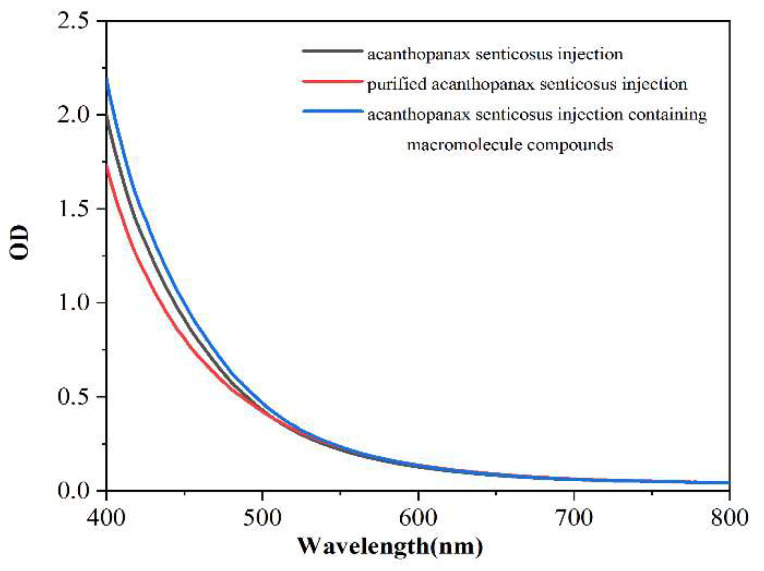
Thermal stability absorption curve of injection (15 d).

**Figure 4:**
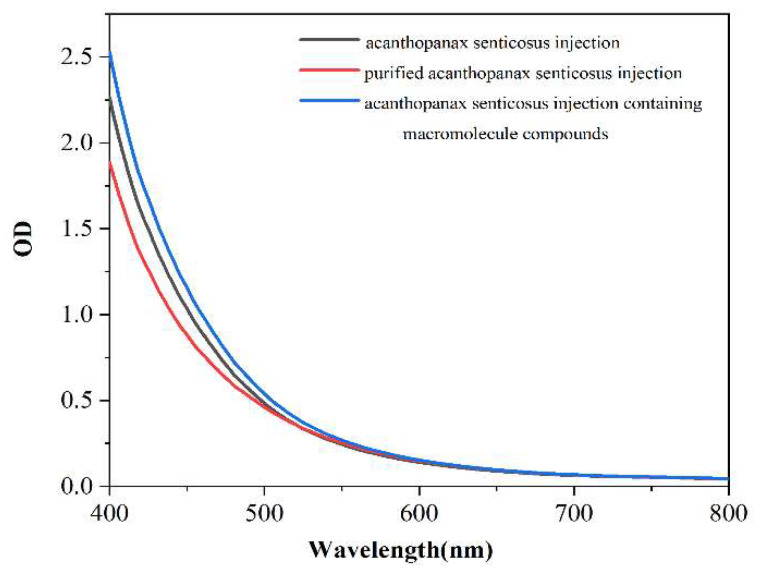
Thermal stability absorption curve of injection (30 d).

**Figure 5:**
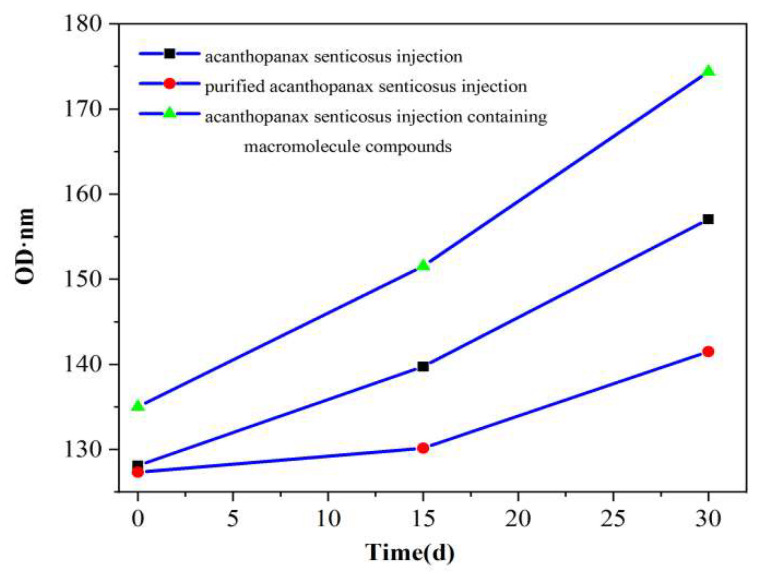
Thermal stability absorption area of injection.

The area under the absorption curve of injection represents the turbidity degree of injection. Figure 2, Figure 3 and Figure 4 shows the thermal stability test results of the original liquid of the acanthopanax senticosus injection, purified acanthopanax senticosus injection, and acanthopanax senticosus injection containing macromolecules at 0, 15, and 30 days. From Figure 2, Figure 3 and Figure 4, compared with the original liquid of the acanthopanax senticosus injection on the first day, the area under the absorption curve for purified acanthopanax senticosus injection decreased, while that of an acanthopanax senticosus injection containing macromolecules increased. As the experiment progressed, the showed rules of the test samples on the 15th and 30th days became increasingly obvious. Especially on the 30th day, the areas under the absorption curves of the original liquid of the acanthopanax senticosus injection, purified acanthopanax senticosus injection, and acanthopanax senticosus injection containing macromolecules were 157.05 OD·nm, 141.49 OD·nm, and 174.37 OD·nm, respectively (Figure 5). The purified acanthopanax senticosus injection had higher clarity and stability than the original solution of the acanthopanax senticosus injection, while the acanthopanax senticosus injection containing macromolecules had higher turbidity and poor stability, which were consistent with the results in Figure 1.

**Figure 6:**
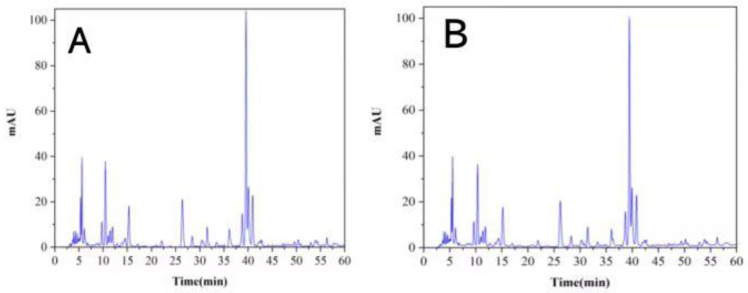
Fingerprints of acanthopanax senticosus injection and purified acanthopanax senticosus injection. Note: (**A**) Fingerprint of the original solution of acanthopanax senticosus injection; (**B**) Fingerprint of purified acanthopanax senticosus injection.

Fingerprints of the original solution of the acanthopanax senticosus injection and purified acanthopanax senticosus injection are shown in Figure 6. By comparison with Figure 6A,B, it can be seen that the fingerprint of the original solution of the acanthopanax senticosus injection was highly similar to the purified acanthopanax senticosus injection; they both had 14 obvious peaks. The peak area of 14 chromatographic peaks was integrated, and the relative peak area of a single peak and total peak for the purified acanthopanax senticosus injection was calculated with the corresponding peak of the original solution of the acanthopanax senticosus injection as reference; the results are shown in Table 1 and Table 2. From Table 1 and Table 2, the relative peak area of each single peak was not less than 0.95, and the relative total peak area was greater than 0.96. As a result, the fingerprint of the acanthopanax senticosus injection purified by PES-C/emoin ultrafiltration membrane was not significantly changed, which showed its active component content did not change.

**Figure 7:**
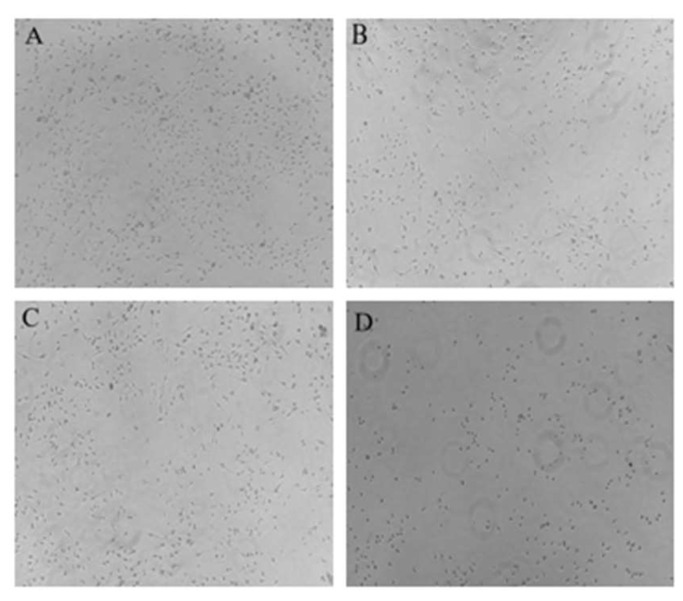
Inhibitory effect of different samples on RBL-2H3 cells. Note: (**A**–**D**) were the blank group, purified acanthopanax senticosus injection group, original solution of acanthopanax senticosus injection group, and acanthopanax senticosus injection containing macromolecules group, respectively.

Photos of the interaction between the original solution of acanthopanax senticosus injection, purified acanthopanax senticosus injection, acanthopanax senticosus injection containing macromolecules (all the injections were diluted to 10% with the medium solution), and the blank group with RBL-2H3 cells are shown in Figure 7. Compared with the original solution of the acanthopanax senticosus injection, the toxicity of the acanthopanax senticosus injection containing macromolecules on RBL-2H3 cells was enhanced; however, the toxicity of the purified acanthopanax senticosus injection weakened. Specifically, IC50 values of the acanthopanax senticosus injection, purified acanthopanax senticosus injection, and the acanthopanax senticosusa injection containing macromolecules by CCK8 method are shown in Table 3, Table 4 and Table 5. From Table 3, Table 4 and Table 5, the ability of cell proliferation decreased with the increase of drug concentration. According to the statistical analysis of SPSS26.0 software, the IC50 of the original solution of the acanthopanax senticosus injection was 11.18%, the IC50 of the purified acanthopanax senticosus injection and the acanthopanax senticosus injection containing macromolecules was 14.41% and 5.02%, respectively.

**Figure 8:**
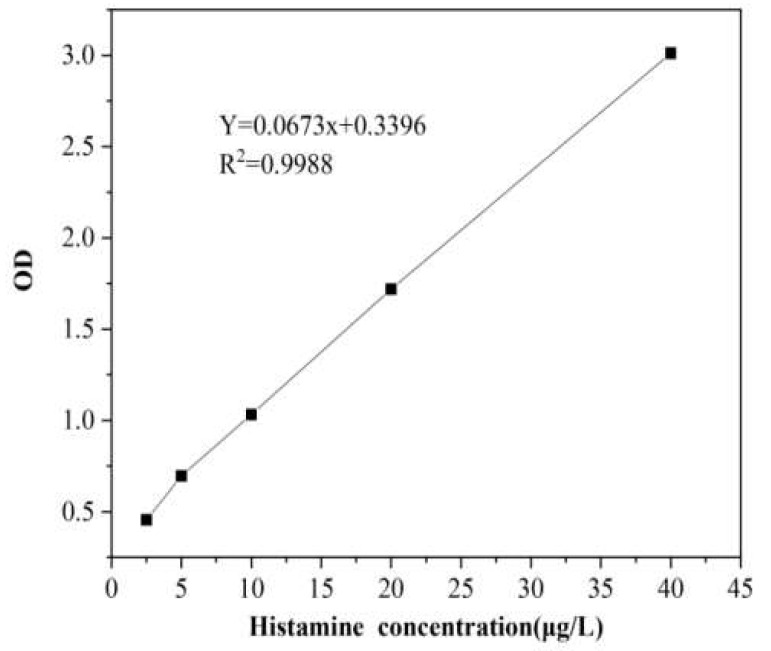
Standard curve for histamine standard substance.

The standard curve of histamine standard is shown in Figure 8. From Figure 8, the standard curve equation of histamine standard is y = 0.0673x + 0.3396, R^2^ = 0.9988, and the concentration of histamine standard is in the range of 2.5–40 μg∙L^−1^. There was a good linear relationship between the histamine concentration and OD value.

According to the standard curve of histamine, the calculated results of histamine concentration in the test samples are shown in Table 6. From Table 6, compared with the original solution of the acanthopanax senticosus injection group, the release amount of histamine from the acanthopanax senticosus injection containing macromolecules significantly increased (*p* < 0.05) when the interaction time with the drug was 10 min and 50 min, and that of the purified acanthopanax senticosus injection was slightly decreased (*p* < 0.05). Compared with the blank group and the original solution of the acanthopanax senticosus injection group, the histamine release amount in the C48/80 group significantly increased (*p* < 0.01). The results suggested that the acanthopanax senticosus injection containing macromolecules was prone to allergic reactions, while purified acanthopanax senticosus injection was safer.

**Figure 9:**
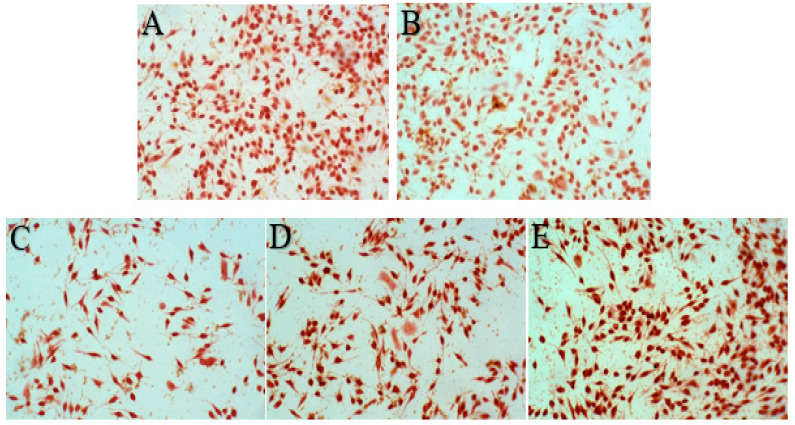
Morphology of RBL-2H3 cells (200×). Note: (**A**–**E**) were the blank group, original solution of acanthopanax senticosus injection group, purified acanthopanax senticosus injection group, acanthopanax senticosus injection containing macromolecules group, and C48/80 group, respectively.

The morphology of RBL-2H3 cells treated with samples is shown in Figure 9. Normal RBL-2H3 cells showed long spindle or pleomorphis with full cytoplasm and complete cell membrane, while the volume of degranulated cells increased, and even the cell membrane burst and the granular materials in the cells released. The results of the cell degranulation rate are shown in Table 7. According to Table 7, compared with the original solution of the acanthopanax senticosus injection group, the cell degranulation rate of purified acanthopanax senticosus injection was slightly lower (*p* < 0.05), the cell degranulation rate of the acanthopanax senticosus injection containing macromolecules increased significantly (*p* < 0.05). As a result, an acanthopanax senticosus injection containing macromolecules easily stimulated RBL-2H3 cells and triggered allergic reactions, while a purified acanthopanax senticosus injection significantly reduced the risk of allergic reactions, which was consistent with the histamine release result in Table 6. The reason was that the acanthopanax senticosus injection containing macromolecules could easily stimulate RBL-2H3 cells to degranulate; thus, histamine, bradykinin, and other allergenic substances were released.

Routine blood test results of the blank group, acanthopanax senticosus injection, and acanthopanax senticosus injection containing macromolecules are shown in Table 8. Five days after the mice were given the drug, compared with the original solution of the acanthopanax senticosus injection, the number of white blood cells, hemoglobin, and platelets in the acanthopanax senticosus injection containing macromolecules increased (*p* < 0.05), because the macromolecular substances activated the relevant inflammatory system. In clinical practice, the increase in the number of white blood cells and platelets often means the chronic infection, tissue damage, and inflammation caused by bacteria and viruses. Compared with the blank group, the number of white blood cells, hemoglobin, and platelets in the acanthopanax senticosus injection group decreased. This was directly related to the effect of the acanthopanax senticosus injection on reinforcing the liver and kidneys. In addition, other detection indexes of the acanthopanax senticosus injection, acanthopanax senticosus injection containing macromolecules, and the blank group showed little change (*p* > 0.05).

Table 9 shows the detection results of organ coefficients in the blank group (normal saline), the original solution of the acanthopanax senticosus group, and the acanthopanax senticosus injection containing macromolecules. From Table 9, compared with the original solution of the acanthopanax senticosus injection, the liver and kidney indexes of the acanthopanax senticosus injection containing macromolecules had no significant difference (*p* > 0.05). However, they increased to a certain extent, indicating that the acanthopanax senticosus injection containing macromolecules had a certain liver and kidney toxicity in mice. In addition, compared with the blank group, the changes in liver and kidney indexes for the original solution acanthopanax senticosus injection group were insignificant (*p* > 0.05).

**Figure 10:**
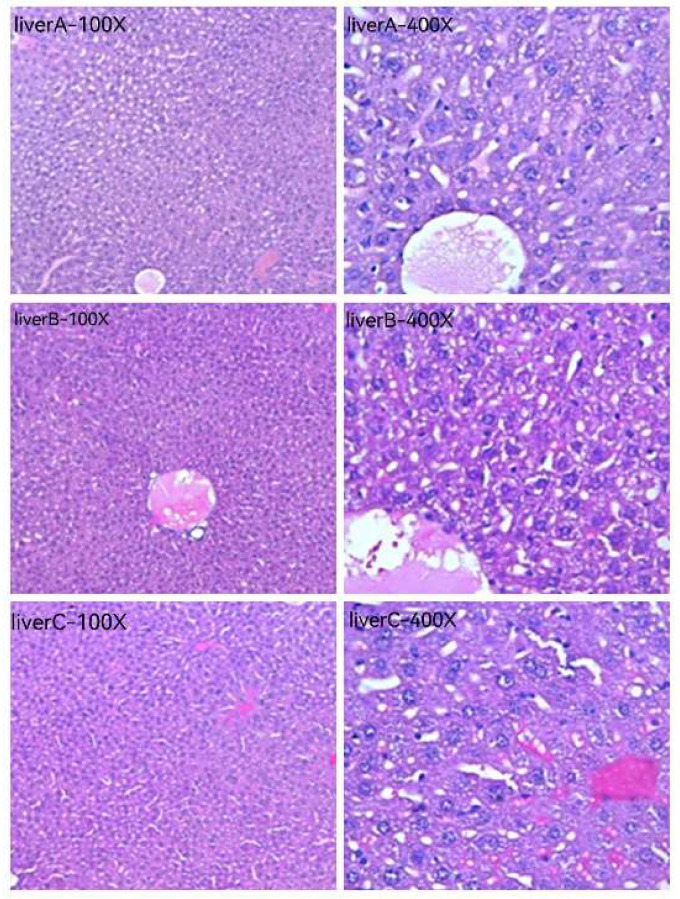
HE staining photos of mice livers using HE staining (100×, 400×). Note: (**A**–**C**) were a blank group, original solution of acanthopanax senticosus injection, and acanthopanax senticosus injection containing macromolecules, respectively.

**Figure 11:**
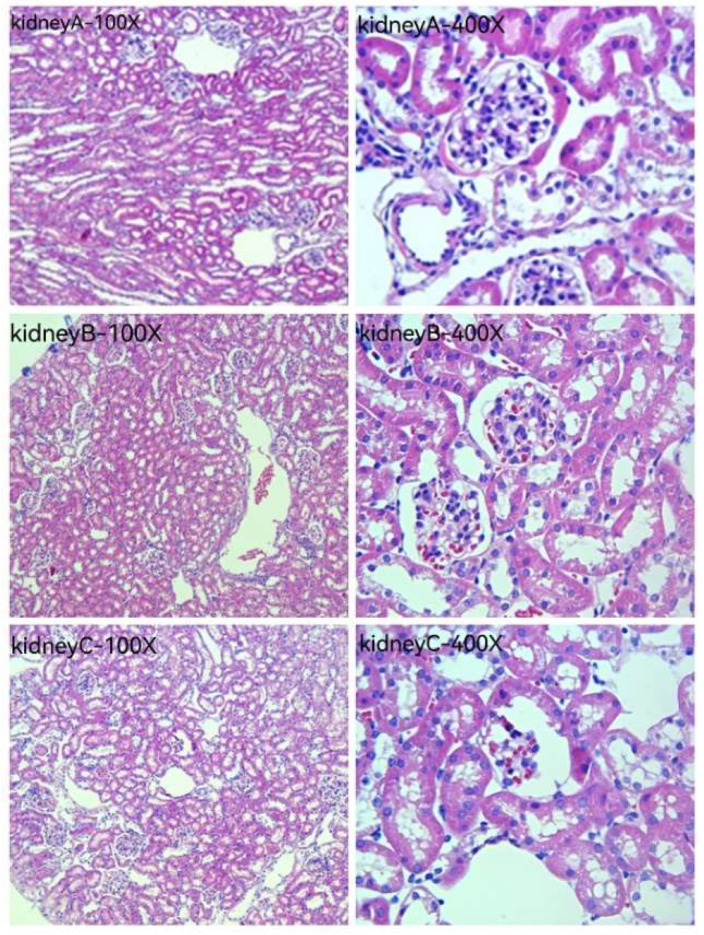
The pathological observation of mice kidneys by HE staining (100×, 400×). Note: (**A**–**C**) were the blank group, original solution of acanthopanax senticosus injection, acanthopanax senticosus injection containing macromolecules, respectively.

Figure 10 and Figure 11 are HE staining photos of the liver and kidney for the blank group (normal saline), the original solution of the acanthopanax senticosus group, and the acanthopanax senticosus injection containing macromolecules. After administration of the original solution of the acanthopanax senticosus group and the acanthopanax senticosus injection containing macromolecules, the microscopic pathological changes of the liver and kidneys were observed in each group. The pathological changes of the acanthopanax senticosus injection containing macromolecules were more serious. In Figure 10, liver A (blank group): the liver tissue section showed a completely normal hepatic lobule structure with radial central vein, there were no obvious changes in hepatic cord and hepatic sinusoid boundary and no cell infiltration around the central vein, and the nuclear position was clear. Liver B (original solution group of acanthopanax injection): hepatic lobules were normal in shape, hepatic cords were neatly arranged, and some nuclei were clearly located, while a small number of cells were degenerated, and inflammatory cells in the central venous area were lightly infiltrated. Liver C (acanthopanax injection group rich in macromolecules): the morphology of hepatic lobules changed with the occasional false lobules, hepatic cords were disorganized, the boundary of hepatic cords and hepatic sinus was unclear, nuclei were necrotic and fused into pieces, and a large number of inflammatory cells were infiltrated in portal duct area and central venous area. Kidney A (blank group): renal tissue section showed no hyperplasia of glomerulus distributed in the cortex, the intact structure of renal tubular epithelial cells, and normal morphology of renal interstitial cells. Kidney B (original solution group of acanthopanax injection): edema for the cortical glomerular epithelial cells was slightly absorbed, the renal tubular cells had slight congestion, and the infiltrated inflammatory cells and congestion were mild. Renal C (acanthopanax injection group rich in macromolecules): the glomerular epithelial cells distributed in the cortex had mild edema, the renal tubules had congestion, and there were infiltrated inflammatory cells and partial congestion in the interstitial cells.

## 3. Experiments

### 3.1. Preparation of PES-C Ultrafiltration Membrane Composited with Emodin

PES-C/emodin ultrafiltration membrane was prepared by the phase transformation method according to the previous experiment method [20]. A certain quality of PES-C was dissolved in a certain volume of N-methylpyrrolidone (NMP) solvent, and the mixture system was stirred at 75 °C for 3 h. After the temperature dropped to room temperature, an emodin of 0.015 wt. % was added into the above system under stirring conditions until a stable, uniform, and transparent casting membrane solution was formed. The casting membrane solution was stood for 12 h and defoamed, and then poured onto a clean glass plate and placed in the air for 15 s. An adjustable film scraper was used to scrape the membrane with a thickness of 200 μm. The glass plate containing the casting membrane solution was immersed in 25 °C deionized water to transform the phase into a film. After the membrane fell off, it was moved to 50 °C deionized water to solidify the pore structure.

### 3.2. Physical Property Experiment of Purified Acanthopanax Senticosus Injection

#### 3.2.1. Preparation and Appearance Color Detection of Purified Acanthopanax Senticosus Injection

The self-made PES-C/emodin ultrafiltration membrane was soaked in PBS solution. The terminal filtration method was used to purify the acanthopanax senticosus injection, and the purified acanthopanax senticosus injection was obtained. Meanwhile, acanthopanax senticosus injection containing macromolecules was obtained (acanthopanax senticosus injection containing macromolecules: purified acanthopanax senticosus injection = 1:19). The obtained samples were stored at −4 °C for later use. The whole experiment was conducted under the aseptic condition. A 10 mL purified acanthopanax senticosus injection, a 10 mL acanthopanax senticosus injection containing macromolecules, and a 10 mL acanthopanax senticosus injection were placed in a sterile test tube with a lid, and their changes in appearance and color were observed and recorded.

#### 3.2.2. Thermal Stability Test of Purified Acanthopanax Senticosus Injection

A 10 mL purified acanthopanax senticosus injection, 10 mL acanthopanax senticosus injection containing macromolecules, and 10 mL acanthopanax senticosus injection were placed in a sterile test tube with a lid and placed in a 60 °C for different times. The above samples were taken out at 50 μL per time on the first day, the 15th day, and the 30th day and added into the transparent 384 well plate. The area under the absorption curve from the enzyme standard instrument determined the overall change of the sample color. The absorption value at each wavelength for the absorption curve was summed to obtain the area under the absorption spectrum curve, and the unit was OD nm.

#### 3.2.3. Fingerprint Detection of Purified Acanthopanax Senticosus Injection

Acetonitrile water (30:70) was used as mobile phase A, 0.5% formic acid solution was used as mobile phase B, and a gradient elution was performed according to Table 10. The detection wavelength was 270 nm, the column temperature was 20 °C, and the flow rate was 0.8 mL·min^−1^. A 10 μL original solution of acanthopanax senticosus injection was filtered by a 0.45 μm nylon filter and diluted by 10 times, and a 10 μL purified acanthopanax senticosus injection was injected into high-performance liquid chromatography (HPLC). Liquid chromatographic images between 0–60 min were recorded, and the changes in relative peak area and total peak area of each fingerprint peak were calculated. Three parallel experiments were conducted for each sample.

#### 3.2.4. Statistical Analysis

SPSS26.0 was used for the statistical treatment, and the measurement results were expressed as mean ± standard deviation ($\bar{X}\pm S$).

### 3.3. Evaluation of Allergic Reaction of Purified Acanthopanax Senticosus Injection

#### 3.3.1. Determination of IC50

Cell suspension density of RBL-2H3 cells at the logarithmic phase was adjusted to 1.0 × 10^5^/mL, and the cells were inoculated into a 96-well plate at the rate of 100 μL/well. PBS was added to the surrounding wells to eliminate the edge effect. Cells were cultured at 37 °C, 5%CO_2,_ and completely saturated with humidity until the cells adhered to the wall, and the culture medium was discarded. The experiment included a blank group (culture medium of 15% fetal bovine serum), experimental group (1) (original solution of acanthopanax senticosus injection: diluted to 5%, 10%, 20%, 30%, 40%, and 50% with the medium solution), experimental group (2) (purified acanthopanax senticosus injection: diluted to 5%, 10%, 20%, 30%, 40%, and 50% with the medium solution), and experimental group (3) (acanthopanax senticosus injection containing macromolecules: being diluted to 5%, 10%, 20%, 30%, 40%, and 50% with the medium solution). Three multiple wells/each group and 100 μL/each well were set. A 96-well plate was placed in the incubator for 24 h at 37 °C, then the medium containing the drug in the well plate was sucked out, washed with PBS 2–3 times, and then the normal EMEM medium was added again. At the same time, 10 μL CCK-8 solution (CCK-8:EMEM = 1:9) was added to each well. CCK8 solution was added into the blank hole as a blank group to eliminate the influence of CCK8. The cells were incubated at 37 °C, 5%CO_2,_ and full saturation humidity for 1 to 4 h. The absorbance of each well at 450 nm was detected by a microplate reader. The inhibition rate of cell growth was calculated according to Equation (1), and IC50 was calculated using SPSS26.0.

(1) $$\mathrm{Cell}\;\mathrm{inhibition}\;\mathrm{rate}=\frac{\mathrm{OD}\;\mathrm{value}\;\mathrm{of}\;\mathrm{cells}\;\mathrm{in}\;\mathrm{control}\;\mathrm{group}-\mathrm{OD}\;\mathrm{value}\;\mathrm{of}\;\mathrm{cells}\;\mathrm{in}\;\mathrm{experimental}\;\mathrm{group}}{\mathrm{OD}\;\mathrm{value}\;\mathrm{of}\;\mathrm{cells}\;\mathrm{in}\;\mathrm{control}\;\mathrm{group}-\mathrm{OD}\;\mathrm{value}\;\mathrm{of}\;\mathrm{cells}\;\mathrm{in}\;\mathrm{blank}\;\mathrm{group}}$$

#### 3.3.2. Determination of Histamine Release

RBL-2H3 cells were inoculated in a 96-well plate according to the ‘Section 3.3.1’. Four groups were set up in the experiment, blank group (culture medium of 15% fetal bovine serum), experimental group 1 (original solution of acanthopanax senticosus injection, final concentration was IC50), experimental group 2 (purified acanthopanax senticosus injection, final concentration was IC50), experimental group 3 (acanthopanax senticosus injection containing macromolecules, final concentration was IC50), and compound 48/80 group (C48/80, 10 μg∙mL^−1^), the last one was served as the positive control group. There were 3 multiple wells and 100 μL in each group. After 10 min and 50 min of interaction between the drug and cells at 37 °C, the supernatant was absorbed and loaded into a sterile 1.5 mL EP tube, centrifuged at 3000 r∙min^−1^ for 20 min, and the supernatant was sucked out again. Other procedures should strictly follow the methods provided in the instructions of the rat histamine ELISA kit. According to the linear regression equation of the histamine standard curve, the sample concentration was calculated through the sample OD value was substituted into the equation, and then multiplied by the dilution factor, the actual concentration of the sample was obtained.

#### 3.3.3. Determination of Cell Degranulation Rate

RBL-2H3 cells were inoculated in a 96-well plate. The experiment included a blank control group, original solution of acanthopanax senticosus injection group, filtrate of acanthopanax senticosus injection group, acanthopanax senticosus injection containing macromolecules group, and C48/80 group (positive group, 10 μg∙mL^−1^), and three multiple wells were set in each group. After cultivation for 30 min, the supernatant was sucked out, 0.5% neutral red dye of 400 μL was added to each well, and the staining lasted for 0.5–1.0 h. Cells were observed under an inverted microscope, and the number of degranulated cells in 100 cells for each well was calculated. The percentage of degranulated cells = (the degranulated cells number/the total cells number) × 100%.

#### 3.3.4. Statistical Analysis

SPSS26.0 was used for the statistical treatment, and the differences among each group were analyzed by a single factor. LSD test was used to compare the mean values of each experimental group, and the measurement results were expressed as mean ± standard deviation ($\bar{X}\pm S$), *p* < 0.05 was a significant difference, and *p* < 0.01 was a very significant difference.

### 3.4. Subacute Toxicity Test of Acanthopanax Senticosus Injection Containing Macromolecules

#### 3.4.1. Grouping and Administration of Experimental Animals

Thirty mice were randomly divided into three groups: blank group (normal saline), original solution of acanthopanax senticosus injection group, and acanthopanax senticosus injection containing macromolecules group, male and female half. According to the clinical dosage of acanthopanax senticosus injection, the dosage for a mouse was determined to be 0.5 mL. Mice were injected through a tail vein once a day for 5 days.

#### 3.4.2. Routine Blood Test

After 5 days of tail vein administration, the experimental rats were weighed and injected with 3.5% chloral hydrate (5 mL∙kg^−1^) as the abdominal anesthesia. Orbital venous blood was collected. Fresh blood was anticoagulated with an EDTA-K2 anticoagulation tube for routine blood testing (white blood cell, red blood cell, hemoglobin, mean hemoglobin concentration, hematocrit, mean red blood cell volume, red blood cell distribution width, platelet, thrombocytocrit, etc.). The routine blood test was performed by automatic hematology analysis.

#### 3.4.3. Detection of Organ Coefficient

Five days after tail vein administration, the abdominal cavity of the mouse was cut open, liver and kidney tissues were removed and washed in PBS, and residual water was sucked up with the absorbent paper. The organ sample was weighed immediately and used to calculate the organ index (organ index = organ weight/body weight × 100%). After weighing, the organ was fixed in 4% paraformaldehyde and stored at 4 °C for HE detection.

#### 3.4.4. Microscopic Pathological Examination

Hematoxylin-eosin staining: Mice liver and kidney tissues were fixed in 4% paraformaldehyde solution for 24 h, paraffin embedding, sectioning, sticky, and dimethyl benzene dewaxing for 2 times was employed, the graded ethanol was used to hydrate step by step, and dying was performed for 10 min with hematoxylin solution. Each section was stained with 1 drop or 50 μL eosin solution for 30 s, the excess dyeing liquid was washed with deionized water, and the sample was sealed with neutral gum. The staining results and tissue morphology were recorded using a microscope.

#### 3.4.5. Statistical Analysis

SPSS26.0 was used for the statistical treatment, and the differences among each group were analyzed by a single factor. An LSD test was used for the mean comparison between experimental groups, and the measurement results were expressed as mean ± standard deviation ($\bar{X}\pm S$); *p* < 0.05 was a significant difference, and *p* < 0.01 is a very significant difference.

## 4. Conclusions

After the acanthopanax injection was purified using a PES-C/emodin ultrafiltration membrane, the macromolecules content in the acanthopanax injection was reduced, its clarity and stability were improved, and the risks of allergic reaction and liver and kidney toxicity were effectively reduced. The active ingredients of the acanthopanax injection showed no obvious change. This technology effectively improved the safety of the acanthopanax injection.

## Figures and Tables

**Table 1:** Changes of single peak area of injection ($\bar{x}\pm s$, n = 3).

Peak Number	Retention Time (min)	Peak Area of the Original Solution of Acanthopanax Senticosus Injection (mAU·min)	Peak Area of the Purified Acanthopanax Senticosus Injection (mAU·min)	Relative Peak Area of Single Peak
1	5.60	327.60 ± 5.84	323.41 ± 7.46	0.99
2	6.13	125.30 ± 2.59	123.43 ± 4.26	0.99
3	9.74	144.41 ± 3.86	137.00 ± 1.75	0.95
4	10.48	475.41 ± 4.94	454.22 ± 5.29	0.96
5	15.32	313.45 ± 3.50	307.03 ± 3.98	0.98
6	26.39	476.53 ± 5.83	462.47 ± 1.99	0.97
7	28.44	88.01 ± 8.36	83.95 ± 1.76	0.95
8	30.47	88.55 ± 8.11	87.81 ± 7.68	0.99
9	31.55	159.06 ± 8.39	150.86 ± 3.01	0.95
10	36.12	166.99 ± 6.37	163.01 ± 5.31	0.98
11	38.77	252.90 ± 12.34	242.07 ± 10.41	0.96
12	39.58	1557.78 ± 38.62	1483.05 ± 74.41	0.95
13	40.78	472.58 ± 4.40	453.62 ± 5.55	0.96
14	40.94	414.68 ± 3.70	400.29 ± 3.65	0.97

**Table 2:** Changes of total peak area of injection ($\bar{x}\pm s$, n = 3).

Total Peak-Peak Area of Acanthopanax Senticosus Injection (mAU·min)	Total Peak-Peak Area of the Purified Acanthopanax Senticosus Injection (mAU·min)	Relative Peak Area of Total Peak
5148.99 ± 33.55	4956.10 ± 48.41	0.96

**Table 3:** Inhibitory effect of the acanthopanax senticosus injection with different concentrations on RBL-2H3 cells ($\bar{x}\pm s$, n = 3).

Original Solution of Acanthopanax Senticosus Injection (%)	Inhibition Rate (%)
5	34.00 ± 0.52
10	42.16 ± 0.97
20	67.31 ± 0.71
30	71.97 ± 1.15
40	76.23 ± 0.43
50	79.98 ± 1.70
IC_50_	11.18

**Table 4:** Inhibitory effect of purified acanthopanax senticosus injection compound with different concentrations on RBL-2H3 cells ($\bar{x}\pm s$, n = 3).

Purified Acanthopanax Senticosus Injection (%)	Inhibition Rate (%)
5	32.16. ± 0.31
10	38.24 ± 1.20
20	60.13 ± 0.86
30	65.26 ± 0.74
40	67.23 ± 1.39
50	71.98 ± 0.67
IC_50_	14.41

**Table 5:** Inhibitory effect of the acanthopanax senticosus injection containing macromolecule compounds with different concentrations on RBL-2H3 cells ($\bar{x}\pm s$, n = 3).

Acanthopanax Senticosus Injection Containing Macromolecules (%)	Inhibition Rate (%)
5	37.93 ± 0.26
10	80.02 ± 0.89
20	81.72 ± 0.51
30	82.89 ± 1.73
40	83.49 ± 2.20
50	84.16 ± 1.24
IC_50_	5.02

**Table 6:** Histamine emission of RBL-2H3 cells in supernatant ($\bar{x}\pm s$, n = 3).

Group	Histamine Concentration (μg·L^−1^) /10 min	Histamine Concentration (μg·L^−1^) /50 min
Blank group	18.30 ± 1.52	17.48 ± 0.78
Original solution of acanthopanax senticosus injection group	32.77 ± 0.44	49.63 ± 1.83
Purified acanthopanax senticosus injection group	29.72 ± 1.10 *	47.96 ± 0.35 *
Acanthopanax senticosus injection containing macromolecules group	40.67 ± 1.57 *	58.25 ± 1.38 *
C48/80 group	49.57 ± 0.83 ^##^	69.25 ± 1.46 ^##^

Note: Compared with original solution of acanthopanax senticosus group * (*p* < 0.05), compared with blank group ^##^ (*p* < 0.01).

**Table 7:** Degranulation rate of RBL-2H3 cells ($\bar{x}\pm s$, n = 3).

Group	Cell Degranulation Rate (%)
Blank group	10.16 ± 0.26
Original solution of acanthopanax senticosus injection group	13.26 ± 0.34
Purified acanthopanax senticosus injection group	11.75 ± 0.98 *
Acanthopanax senticosus injection containing macromolecules group	19.37 ± 1.46 *
C48/80 group	20.27 ± 0.69 ^##^

+ group ## (*p* < 0.01). Note: Compared with original solution of acanthopanax senticosus group * (*p* < 0.05), compared with blank group ## (*p* < 0.01).

**Table 8:** Effects of acanthopanax senticosus injection containing macromolecule compounds on blood routine in mice ($\bar{x}\pm s$, n = 10).

Detection Index	Blank Group	Original Solution of Acanthopanax Senticosus Injection Group	Acanthopanax Senticosus Injection Containing Macromolecules Group
Hemameba number (10^9^/L)	4.67 ± 0.90	4.60 ± 0.21 ^#^	5.16 ± 0.28 *
Erythrocyte number (10^12^/L)	8.53 ± 0.55	8.02 ± 0.58	8.34 ± 0.62
Hemoglobin content (g∙L^−1^)	133.57 ± 8.56	122.67 ± 30.97 ^#^	133.07 ± 3.71 *
Mean hemoglobin concentration (g∙L^−1^)	301.67 ± 7.23	296.00 ± 15.52	301.30 ± 1.53
Hematocrit (%)	44.17 ± 2.35	41.07 ± 8.64	44.10 ± 1.32
Mean erythrocyte volume (fL)	51.1 ± 0.78	51.87 ± 0.64	52.93 ± 1.97
Erythrocyte distribution width CV (%)	24.67 ± 0.58	24.00 ± 0.00	25.00 ± 1.00
Platelet number (10^9^/L)	566.67 ± 30.60	533.00 ± 87.07 ^#^	600.00 ± 49.15 *
Thrombocytocrit (%)	0.62 ± 0.21	0.57 ± 0.21	0.63 ± 0.46

Note: Compared with the original solution of acanthopanax senticosus injection, * (*p* < 0.05) compared with blank group ^#^ (*p* < 0.05).

**Table 9:** Effects of acanthopanax senticosus injection containing macromolecule compounds on viscera coefficient in mice ($\bar{x}\pm s$, n = 10).

Group	Liver Index (%)	Renal Index (%)
Blank group	4.61 ± 0.42	0.95 ± 0.01
Original solution of acanthopanax senticosus injection group	4.60 ± 0.15	0.94 ± 0.03
Acanthopanax senticosus injection containing macromolecules group	4.73 ± 0.09	0.97 ± 0.12

**Table 10:** Mobile phase concentration in the gradient elution.

Time (min)	Mobile Phase A (%)	Mobile Phase B (%)
0~15	5→12	95→88
15~21	12→18	88→82
21~60	18→69	82→31
60~65	100	0
